# Characterization of Ten Heterotetrameric NDP-Dependent Acyl-CoA Synthetases of the Hyperthermophilic Archaeon *Pyrococcus furiosus*

**DOI:** 10.1155/2014/176863

**Published:** 2014-02-11

**Authors:** Joseph W. Scott, Farris L. Poole, Michael W. W. Adams

**Affiliations:** Department of Biochemistry and Molecular Biology, University of Georgia, Athens, GA 30602, USA

## Abstract

The hyperthermophilic archaeon *Pyrococcus furiosus* grows by fermenting peptides and carbohydrates to organic acids. In the terminal step, acyl-CoA synthetase (ACS) isoenzymes convert acyl-CoA derivatives to the corresponding acid and conserve energy in the form of ATP. ACS1 and ACS2 were previously purified from *P. furiosus* and have *α*_2_ *β*_2_ structures but the genome contains genes encoding three additional *α*-subunits. The ten possible combinations of *α* and *β* genes were expressed in *E. coli* and each resulted in stable and active *α*_2_ *β*_2_ isoenzymes. The *α*-subunit of each isoenzyme determined CoA-based substrate specificity and between them they accounted for the CoA derivatives of fourteen amino acids. The *β*-subunit determined preference for adenine or guanine nucleotides. The GTP-generating isoenzymes are proposed to play a role in gluconeogenesis by producing GTP for GTP-dependent phosphoenolpyruvate carboxykinase and for other GTP-dependent processes. Transcriptional and proteomic data showed that all ten isoenzymes are constitutively expressed indicating that both ATP and GTP are generated from the metabolism of most of the amino acids. A phylogenetic analysis showed that the ACSs of *P. furiosus* and other members of the Thermococcales are evolutionarily distinct from those found throughout the rest of biology, including those of other hyperthermophilic archaea.

## 1. Introduction

Members of the Thermococcales order of the Archaea are anaerobic, heterotrophic microorganisms that grow optimally at or above 80°C [1]. They include species of *Pyrococcus* and *Thermococcus*, all of which utilize peptides as carbon and energy sources and many also ferment carbohydrates [2, 3]. Glucose is metabolized via a modified Emden-Meyerhof pathway that involves ADP- rather than ATP-dependent hexo- and phosphofructokinases [4] and a ferredoxin-linked glyceraldehyde-3-phosphate (GAP) oxidoreductase (GAPOR) rather than an NAD-linked GAP dehydrogenase [5, 6]. The pyruvate that is generated by glycolysis is oxidized by pyruvate ferredoxin oxidoreductase (POR) [7] to yield CO_2_ and acetyl-CoA, and energy is conserved in the form of ATP by an ADP-dependent acetyl-CoA synthetase that generates acetate [8]. The general acyl CoA synthetase reaction is shown in (1). This reaction is in contrast to the two-step conversion in anaerobic bacteria whereby acetyl CoA is transformed to acetate via phosphotransacetylase and acetate kinase:

(1) $$\begin{array}{c} \text{R}.\;\text{CO}.\;\text{SCoA}+\text{ADP}+\text{Pi}⟶\text{RCOOH}+\text{ATP}+\text{CoASH} \end{array}$$

The proposed pathway for peptide fermentation involves the hydrolysis of peptides into individual amino acids followed by their oxidative deamination to 2-keto acids [9]. Further metabolism of the 2-keto acids is analogous to pyruvate oxidation by POR, in which they are oxidatively decarboxylated to the acyl-CoA derivatives that then serve as substrates for ADP-dependent CoA synthetases. This involves three other ferredoxin-dependent keto acid oxidoreductases. Indolepyruvate ferredoxin oxidoreductase (IOR) and 2-ketoisovalerate ferredoxin oxidoreductase (VOR) oxidize the transaminated products of aromatic amino acids [10] and of branched chain amino acids [11], respectively, while 2-ketoglutarate ferredoxin oxidoreductase (KGOR) oxidizes 2-ketoglutarate, which is derived from glutamate and glutamine [12]. Hence POR is involved in the fermentation of both sugars and peptides (pyruvate is the product of alanine transamination), while IOR, KGOR, and VOR function only in peptide fermentation. The genome of the hyperthermophile *Pyrococcus furiosus* contains two additional ferredoxin-dependent oxidoreductases. XOR is in an operon with KGOR and is coregulated with KGOR at the transcriptional level [13] but the substrate specificity of this fifth 2-ketoacid oxidoreductase is not known. In addition, a homolog composed of the gene products of PF0753 and PF0754 was identified as a protein complex during the fractionation of *P. furiosus* biomass [14]. The substrate specificity of this sixth 2-ketoacid oxidoreductase is also unknown.

ACS therefore catalyzes the terminal step of peptide fermentation, the conversion of acyl CoA derivatives to the corresponding acid with concomitant synthesis of ATP (1). Two forms of ACS, ACS1, and ACS2, have been characterized from *P. furiosus *both in native [8, 15] and recombinant forms [16]. Each consists of two subunits with estimated molecular weights of 45 (*α*) and 25 kDa (*β*) with molecular masses for the holoenzymes of approximately 140 kDa, indicative of *α*_2_ *β*_2_ structures. In addition to acetyl CoA, ACS1 used isobutyryl-CoA as a substrate, while ACS2 used phenylacetyl-CoA and indoleacetyl-CoA. It was therefore proposed that ACS1 was involved in the metabolism of alanine and branched chain amino acids and utilized the products of POR and VOR, while ACS2 played a role in the metabolism of aromatic amino acids via IOR [8]. The two enzymes also differed in their specificity for purine nucleotides as ACS1 preferred GDP/GTP to ADP/ATP while ACS2 preferred ADP/ATP to GDP/GTP [8]. From their N-terminal sequences it was evident that ACS1 and ACS2 did not share any subunit [8] and from the *P. furiosus *genome sequence [17] it was evident that ASC1*α* (PF1540), ACS1*β* (PF1787), ACS2*α* (PF0532), and ACS2*β* (PF1837) are encoded by unlinked genes.

The product of the KGOR reaction is succinyl CoA but no ACS-type activity was detected in extracts of *P. furiosus *cells using this as a substrate [8]. However, an ADP-dependent succinyl-CoA synthetase activity was purified from *Thermococcus kodakarensis*, a member of the Thermococcales and a close relative of *P. furiosus *[18]. This enzyme, which will be referred to here as ACS3, also had an apparent *α*_2_ *β*_2_ subunit stoichiometry. The gene encoding the *α* subunit of *T. kodakarensis* ACS3 has a close homolog in the *P. furiosus *genome (PF0233) while that encoding the *β*-subunit corresponded to PF1837, which encodes the *β*-subunit of *P. furiosus *ACS2. Hence this *β*-subunit is associated with two *α*-subunits to give either *P. furiosus *ACS2 or ACS3. Further inspection of the *P. furiosus *genome reveals that, while there are only two genes that encode ACS *β*-subunits (PF1837 and PF1787), two additional *α*-subunits are present encoded by PF1085 and PF1838. These will be designated as ACS4*α* and ACS5*α*, respectively. Recently, homologs of these two types of ACSs in *P. furiosus *were characterized from *T. kodakarensis* [19]. It was reported that one of them (TK2127) was specific for 2-(imidazole-4-yl)-acetate, a degradation product of histidine fermentation, while the other (TK0944) had a broad substrate range similar to that of *P. furiosus *ACS2. However, in the *T. kodakarensis* study, TK2127 and TK0944 were characterized with only one of the two possible *β*-subunits (TK0943) and only using adenine nucleotides.

*P. furiosus *and other members of the Thermococcales therefore have the genetic potential to synthesize up to ten ACS isoenzymes, depending upon which of the five *α*-subunits associates with which of the two *β*-subunits. The corresponding enzymes will be designated herein as ACSx-A or ACSx-G, where A (PF1837) and G (PF1787) represent the two *β*-subunits encoded by the indicated genes, while *x* (*x* = 1–5) represents the *α*-subunits encoded by PF1540 (1), PF0532 (2), PF0233 (3), PF1085 (4), and PF1838 (5), respectively. The two *β*-subunits are 59% identical at the amino acid sequence level. Hence the two ACS isoforms that have been purified from *P. furiosus *biomass correspond to ACS1-G and ACS2-A, while a third form, ACS3-A, is evident by analogy with the *T. kodakarensis *enzyme. Hence this analysis begs the questions of whether the *α*-subunits in these three forms are able to form catalytically active complexes with the alternate *β*-subunit, which *β*-subunits are associated with ACS4 and ACS5, and what are the substrate specificities of all of these complexes in terms of both acyl-CoA derivatives and adenine and guanine nucleotides. Herein we have addressed these questions by generating the recombinant forms of the ten possible ACS complexes in *P. furiosus *and assessing their preferences for substrates corresponding to all twenty amino acids.

## 2. Materials and Methods

### 2.1. Production of Recombinant ACSs

The genes encoding the five *α*-subunits were amplified by PCR from *P. furiosus *genomic DNA with the following primer sets: PF0233 (ACS3), 5′-AATTTGACATATGACAGTTAACCTAGACTTTC-3^'^ (+) and 5′-CCGCTCGAGTTAGAGCTCAGCTAAATACTTTC-3^'^ (−). PF0532 (ACS2), 5′-AATTTGACATATGCTTGACTACTTCTTTAATCCAAG-3^'^ (+) and 5′-CCGCTCGAGTTAACCATTTACCCCACCTCCAACATTC-3^'^ (−). PF1085 (ACS4), 5′-AATTTGACATATGAGGTACTTCTTTTACCCAAATAG-3^'^ (+) and 5′-CCGCTCGAGTCATTCGCTACTAGACCTTAGGCTGAG-3^'^ (−). PF1540 (ACS1), 5′-AATTTGACATATGAGTTTGGAGGCTCTTTTTAATC-3^'^ (+) and 5′-CCGCTCGAGTTACTTTTCTTTGTGTTTTGCTTTC-3^'^ (−). PF1838 (ACS5), 5′-AATTTGACATATGATTAACAACTTGGACATTAAAG-3^'^ (+) and 5′-CCGCTCGAGTCATTCCCCCATCTTCCTCAAATATTC-3^'^ (−). NdeI and XhoI restriction sites are underlined for the + and − strands respectively. The resulting DNA fragments encoding the *α* subunits were each inserted into the pET24a(+) Kan^r^ expression vector (Novagen, Madison, WI). The genes encoding the two *β* subunit homologs were also amplified from the *P. furiosus *genomic DNA with the following primers: PF1787 (*β*-G), 5′-GATGCCATGGACAGGGTTGCTAAGGCTAGGG-3^'^ (+) and 5′-TAATTTGAGCGGCCGCCTAAAGAATCATCCTAGC-3^'^ (−). PF1837 (*β*-A), 5′-AATTTGACATATGATTAACAACTTGGACATTAAAG-3^'^ (−). and 5′-CCGCTCGAGTCATTCCCCCATCTTCCTCAAATATTC-3^'^ (−). NcoI and NotI restriction sites are underlined for the + and − strands respectively, for the PF1787 DNA and NdeI and XhoI restriction sites underlined for the + and − strands respectively, for the PF1837 DNA. The amplified PF1787 DNA fragment was inserted into pET21d Amp^r^ expression vector and the amplified PF1837 DNA fragment was inserted into pET21b Amp^r^ expression vector. The sequence of each inserted cassette was confirmed by the Sanger method. The *α* subunit expression plasmids (pET24a(+):PF0233, pET24a(+):PF0532, pET24a(+):PF1085, pET24a(+):PF1540, and pET24a(+):PF1838) were cotransformed with each *β*-subunit expression plasmid (pET21d:PF1787 and pET21b:PF1837) into *E. coli* BL21(DE3) pRIL.

For recombinant protein production, cultures were grown with the appropriate antibiotics at 37°C in 250 mL 2xYT media to an optical density between 0.8 and 1.0 at 600 nm. Expression of the recombinant genes was induced by the addition of 0.4 mM IPTG (isopropyl-*β*-D-thiogalactopyranoside) at 37°C. Following 3 hours of induction, the cultures were harvested by centrifugation. The pelleted cells were resuspended in lysis buffer (20 mM bis-Tris, pH 7.0, containing 150 mM NaCl, 10 mM MgCl, 1 mM PMSF (phenylmethanesulfonylfluoride), 0.27 mg/mL lysozyme and 0.007 mg/mL DNAse I) and incubated at room temperature for 30 minutes. After centrifugation at 13,000 ×g for 20 min, the supernatant was heated at 80°C for 15 min and centrifuged to remove precipitated heat-labile *E. coli* proteins. The supernatant was applied to a 10 mL HiTrap desalting column (made by connecting two 5 mL columns in tandem) (GE Healthcare) that had been equilibrated with desalting buffer (20 mM bis-Tris, pH 7.0 containing 150 mM NaCl and 10 mM MgCl). The resulting enzyme preparations were used for substrate specificity analyses. Protein concentrations were determined by the Bradford method [20].

To prepare sample for molecular weight determination, the supernatant from the heat treatment step described above (500 *μ*L, ~1.5 mg protein) was diluted 10-fold in buffer A (20 mM bis-Tris, pH 7.0 containing 2 mM dithiothreitol (DTT) and 5% glycerol) and applied to a 1 mL Mono Q column (GE Healthcare) that had been equilibrated with buffer A. The ACS was eluted with a 20 mL linear gradient from 0 to 0.3 M NaCl in buffer A. Fractions (1 mL) were collected and those containing the ACS as determined by SDS electrophoresis were combined and concentrated to a volume of 200 *μ*L in an Amicon Ultra centrifugation device having a cut-off of 30 kDa. This was applied to a SX200 10/30 (24 mL) gel filtration column that had been previously equilibrated with 50 mM potassium phosphate containing 200 mM KCl, 5% glycerol, 2 mM DTT, and pH 7.0 and calibrated with standard proteins *β*-amylase (200 kDa), alcohol dehydrogenase (150 kDa), BSA (66 kDa), carbonic anhydrase (29 kDa), and cytochrome c (12.5 kDa).

### 2.2. High-Throughput Enzyme Assays

Since the CoA-forms of the 2-keto acids from all 18 amino acids (assuming that glutamate/glutamine and aspartate/asparagine represent two amino acids) are not commercially available, substrate specificities of each ACS isoenzyme was measured using the reverse reaction of (1) using 18 different acid substrates. A discontinuous assay in which phosphate release from the NTP was quantitated was used in a 96-well format. The ACS sample was that obtained after the heat treatment step described above. The 50 *μ*L reaction mixture contained 20 mM bis-Tris buffer, pH 7.0, 0.5 *μ*g ACS, 20 mM acid substrate, 1 mM ATP or GTP, 2.0 mM MgCl_2_, and 0.5 mM coenzyme-A. It was heated at 80°C for 1 minute in a 96-well heating block. The reaction was stopped by adding 1.0 N H_2_SO_4_. The reaction was found to be linear over at least a 2 min assay period. Free phosphate produced by the hydrolysis of NTP was measured using the BioVision colorimetric assay kit (Mountain View, CA). All liquid handling was carried out robotically (Beckman Coulter Biomek FX, Brea, CA). Kinetic parameters were determined by the same method using NTP substrate concentrations over the range from 0.0625 mM to 4.0 mM. The 96-well plate format was also used to assay activity in the forward direction (1) with the appropriate acyl-CoA derivative. The reaction mixture (50 *μ*L) contained acyl-CoA (0.031 mM to 2.0 mM), K_2_HPO_4_ (10 mM), MgCl_2_ (2 mM), ADP (2 mM), and ACS1 (0.5 *μ*g) in 20 mM bis-Tris buffer (pH 7.0). After heating to 80°C for 90 sec, the free CoASH released was measured by the addition of 200 *μ*L of 5′5′-dithiobis(2-nitrobenzoic acid) (DTNB) (0.4 mM) in 100 mM K_2_PO_4_, pH 7.2.

## 3. Results and Discussion

**Figure 1:**
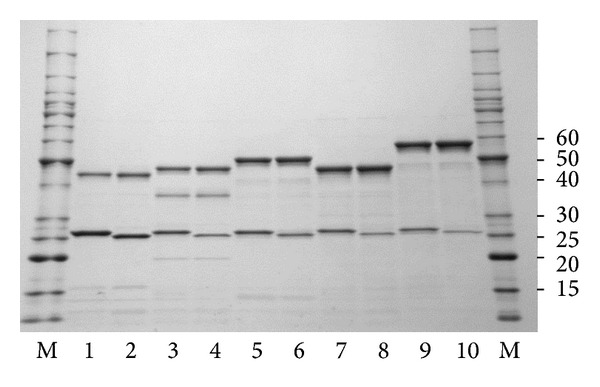
SDS-PAGE analyses of recombinant *P. furiosus* ACS isoenzymes. The lanes correspond to the following: 1: ACS1-G; 2: ACS1-A; 3: ACS2-G; 4: ACS2-A; 5: ACS3-G; 6: ACS3-A, 7: ACS4-G; 8: ACS4-A; 9: ACS5-G; and 10: ACS5-A. Each lane contains approximately 2 *μ*g of the protein obtained after the heat-treatment step. Lanes M indicate molecular weight markers (given in kDa).

Previously ACS1-G of *P. furiosus *was purified from native biomass [8] and the recombinant forms of the two subunits (ACS1-*α* and *β*-G) generated separately in *E. coli *were combined *in vitro *to give a heterotetrameric enzyme that was indistinguishable from that purified from *P. furiosus* [16]. It was unclear, however, if production and combination of individual subunits would be successful for all possible ACS isoenzymes, particularly if cotranslation was required for efficient heteromeric complex formation. Since the *T. kodakarensis* homologs of the *P. furiosus* ACS2-A and ACS3-A isoenzymes (which contain the same *β* subunit) were produced in *E. coli *using a single plasmid encoding both subunits [18], a similar coexpression approach was used herein. Five plasmids conferring kanamycin resistance were constructed which also encoded each of the five *α*-subunits, and two plasmids were constructed conferring ampicillin resistance which also encoded each of the *β*-subunits. Each plasmid encoding a *β*-plasmid was then transformed into *E. coli *with a plasmid encoding one of the *α*-subunits to create all ten possible combinations. Heat treatment of the cell-free extracts of each *E. coli* strain gave rise to two major proteins as determined by SDS PAGE with sizes corresponding to those predicted from the gene sequences of the larger (*α*, 47.8–51.8 kDa) and smaller (*β*, 25.8–26.4 kDa) ACS subunits, as shown in Figure 1. To determine which of the *α*- and *β*-subunit combinations assembled into heteromeric structures, each was analyzed by gel filtration chromatography. All ten eluted with estimated molecular weights of approximately 140 kDa and there was no evidence of individual monomers indicating that all formed stable *α*_2_ *β*_2_ structures when their respective genes were coexpressed in *E. coli*.

**Figure 2:**
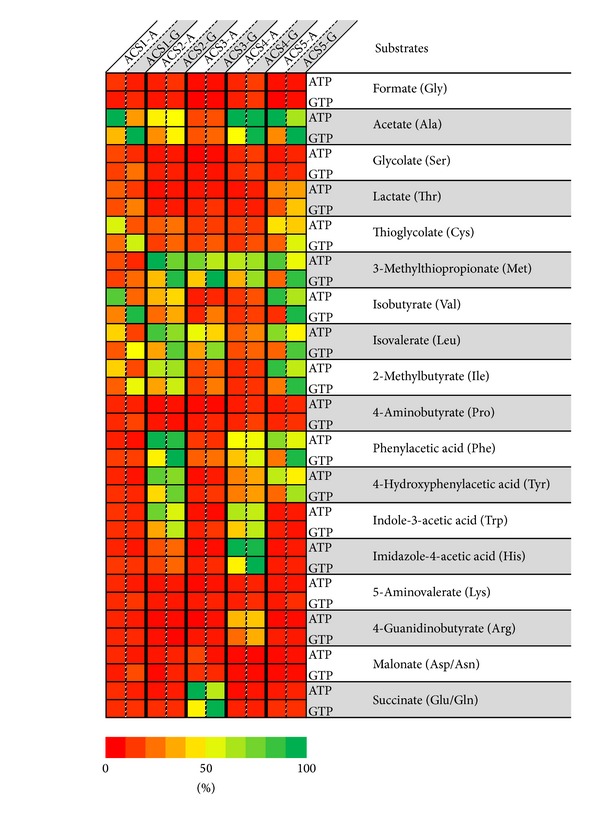
Heat map of activities of the ten ACS isoenzymes with eighteen organic acids with ATP or GTP. 100% activity corresponds to the highest activity of an individual enzyme with ATP or GTP. All other activities in a given column are relative to the highest value.

The substrate specificities of ten recombinant ACS isoenzymes were determined at 80°C using a 96-well plate approach and the eighteen organic acid substrates that correspond to the twenty amino acids (the same acids are generated by glutamate and glutamine, and by aspartate and asparagine) with either ATP or GTP as the co-substrate. A heat map representing the relative activity of each isoform is shown in Figure 2, where 100% activity represents the highest measured value with either ATP or GTP. A lower limit was arbitrarily set at 30% of the specific activity produced by the most active substrate and this was used to establish the substrate specificity of each enzyme. As anticipated, ACS1 was most active with acids derived from alanine and the branched chain amino acids, but surprisingly, it also had significant activity with thioglycolate, the end product of cysteine fermentation. In agreement with previous results [8], ACS2 had overlapping specificity with ACS1 activity but, unlike ACS1, it was also highly active with substrates representing the aromatic amino acids. Surprisingly, ACS2 was also active with 3-methylthiopropionate, the end product of methionine fermentation. Hence ACS1 and ACS2 in combination show reasonable activity with acids derived from 9 of the 20 amino acids. One of the exceptions is succinate, derived from glutamate and glutamine via 2-ketoglutarate. The only isoenzyme that showed activity with succinate was ACS3 (Figure 2). However, ACS3 had similar activity with the methionine derivative, comparable to that of ACS2.

ACS4 was the only isoenzyme that shows significant activity with imidazole 4-acetate, which is derived from histidine. It is also the only ACS that shows detectable activity with the organic acid derived from arginine (Figure 2). On the other hand, like ACS2, the substrate specificity of ACS4 indicates that this isoenzyme is involved in the degradation of alanine and the aromatic amino acids. ACS5 also shows a broad substrate range similar to that of ACS2, and similarly does not utilize derivatives of methionine or lysine. However, ACS5 is the only enzyme that has significant activity with lactate, which is the end product of the threonine pathway. Considering all ten isoenzymes, there are six amino acids whose acid derivatives were not utilized by any of them and these were glycine, serine, proline, lysine, aspartate, and asparagine.

Of the fourteen acids utilized by the recombinant ACSs in the work presented herein, nine of them (acetate, 3-methylthiopropionate, isobutyrate, isovalerate, 2-methylbutyrate, phenylacetic acid, 4-hydroxyphenylacetic acid, indole-3-acetic acid, and succinate) result from the four KORs that have been characterized [10–12]. However, the genome of *P. furiosus* harbors two additional KORs. The genes for one (XOR, PF1771-PF1773) are expressed and are regulated by carbon source [13] while the genes for the other (PF0753, PF0754) are also expressed and the corresponding proteins have been identified in *P. furiosus *biomass [14]. Furthermore, it is not known if the KORs that have been characterized can use as substrates the 2-keto acid derived from threonine, arginine, cysteine, and histidine. It is therefore reasonable to conclude that the activities of the known and as yet uncharacterized KORs account for the ACS activities described herein for *P. furiosus* and also for *T. kodakarensis* [19]. On the other hand, the biosynthetic pathways for amino acids in *P. furiosus* appear to be generally similar to the well-studied routes in mesophilic bacteria. For example, DNA microarray analyses were consistent with the biosynthesis of twelve amino acids (Glu, Arg, Leu, Val, Ile, Ser, Thr, Met, His, Phe, Trp, and Tyr) occurring by conventional pathways [13]. However, growth studies to determine amino acid auxotrophy have given ambiguous results [21–23] and while the genome of *P. furiosus *does encode genes that could be involved in the biosynthesis of all twenty amino acids, some pathways for amino acids may be incomplete [24]. A more comprehensive understanding of amino acid metabolism in *P. furiosus* in general is clearly needed before a more definitive correlation between biosynthetic and degradative amino acid pathways can be made and the corresponding roles of the ACS isoenzymes.

Using the 96-well plate approach, *K*_*m*_ values for ATP and acetate of 0.34 and 1.6 mM, respectively, were determined at 80°C for recombinant ACS1-G. These values are similar to those reported for the same ACS isoenzyme purified from *P. furiosus* native biomass [8], values that were obtained using the same assay conditions as used herein but with one at a time measurements. However, the values are at least two-fold higher than those previously reported for the recombinant ACS-G isoenzyme (0.090 and 0.8 mM, resp. [25]), although those data were based on a coupled assay at 55°C, which might account for the differences. From the data presented in Figure 2 it is evident that all five ACS *α*-subunits utilize acetyl CoA (forward reaction of (1)) and the kinetic constants for each ACS-A enzyme is given in Table 1. The *K*_*m*_ and the *k*_cat_/*K*_*m*_ values are quite similar for ACS1, ACS2, ACS4 and ACS5, with ACS3 having comparable affinity but much lower activity. Hence all four isoenzymes likely utilize acetyl CoA *in vivo*. Similarly, the results indicate that ACS2 and ACS5 are comparably active with CoA derivatives derived from branched chain amino acids (Table 1). Unfortunately, the primary substrate for ACS3, succinyl-CoA, could not be used at 80°C as it is extremely unstable at the assay temperature and direct comparisons were not possible. Similarly, the primary CoA substrates for ACS4 and ACS5, derived from histidine and threonine, are not commercially available.

It is interesting to compare the results described herein with *P. furiosus* to those recently reported with *T. kodakaraensis* by Shikata et al. [18] and Awano et al. [19]. For example, while the substrate specificities for the ACS3 homolog from each organism were very similar, *P. furiosus* ACS4 utilized acids derived from histidine, methionine, phenylalanine, tryptophan and arginine, while its counterpart in *T. kodakaraensis* was only reactive with the histidine derivative. Similarly, in *P. furiosus *the substrate range of ACS5 is extended compared to that of the *T. kodakaraensis *homolog as it was also active with lactate, a derivative of threonine fermentation. While kinetic comparisons are limited since the *T. kodakaraensis *homologs were studied mainly using acids as the substrates, in both studies ACS3 exhibited Michaelis-Menten kinetics when acetyl CoA and ADP were used as substrates. However, the *T. kodakaraensis* enzyme had a much lower *K*_*m*_ value (41 *μ*M) and higher catalytic efficiency (0.460 *μ*M^−1^·s^−1^) than *P. furiosus* ACS3 (474 *μ*M and 0.012 *μ*M^−1^·s^−1^). It is not clear to what extent this reflects physiological differences between the two organisms.

The specificities for the organic substrates of the ACS isoenzymes of *P. furiosus *containing each of the five *α*-subunits was independent of whether they were associated with the *β*-A or *β*-G subunits (Figure 2). However, the type of *β* subunit did determine the nucleotide specificity. With all ten isoenzymes, those containing the *β*-A subunit were more active with ATP than GTP. In contrast, enzymes containing the *β*-G subunit were generally more active with GTP than ATP or at least showed comparable activity with ATP and GTP (Figure 2). This was further demonstrated by the kinetic parameters of ACS1-G and ACS1-A with GTP and ATP as substrates. The catalytic efficiency of ACS1-A was approximately 14-fold higher with ATP than with GTP, while the opposite was true with ACS1-G (Table 2). Hence the nucleotide specificity of the ACS isoenzymes is determined by the *β*-subunit. This is consistent with the presence of two ATP-grasp fold subdomains in the *β*-subunits (residues 23–139 in A*β* and 25–139 in G*β*), one of which includes a conserved histidinyl residue (His71) at the active site [26]. *P. furiosus* ACSs presumably transfer the phosphate group from the *α*-subunit to this conserved histidine of the G-*β* subunit, which then transfers the phosphate group to the nucleoside diphosphate [26].

Hence, the ten ACS isoenzymes in *P. furiosus *can account for the metabolism of the majority (14 of 20) of amino acids, using both adenine and guanine nucleotides. The ACS reaction is the only means of conserving energy during peptide degradation, and the same is true for sugar fermentation. This is due to the unusual modification of the glycolytic pathway in *P. furiosus, *where GAP oxidation is coupled to the reduction of ferredoxin instead of NAD, resulting in the conservation of energy at the ferredoxin oxidation step via a respiratory membrane-bound hydrogenase [27, 28] rather than by substrate level phosphorylation (SLP) via 1,3-bisphosphoglycerate [3, 5]. Hence the ACS reaction generating acetate is the only source of ATP via SLP. The results presented herein show that all five ACS-A isoenzymes appear to be efficient in catalyzing this reaction, as well as being involved in peptide degradation. Since ATP is the principle energy carrier within the cell, it would be expected that all ACS-A isoenzymes (containing the *β*-A subunit) would catalyze these reactions. It is therefore not obvious what advantage is gained by using the *β*-G subunit to produce GTP. It would seem futile for an NDP kinase to transphosphorylate ADP with the GTP since ATP is made via ACS isoenzymes with the *β*-A subunit.

**Figure 3:**
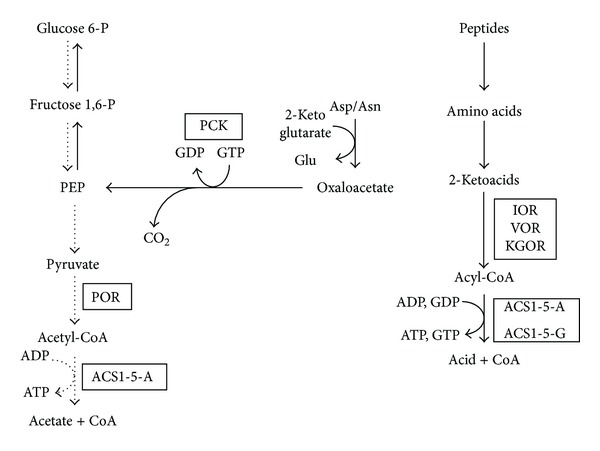
Proposed function of ACS *β*-G and ACS *β*-A isoenzymes during growth of *P. furiosus *on carbohydrates or peptides. The abbreviations are: POR, pyruvate ferredoxin oxidoreductase; KAOR, 2-keto acid oxidoreductases (IOR, VOR, KGOR and POR, see text for details).

Insight into the function of ACS isoenzymes containing the *β*-G subunit is provided by Fukuda and coworkers [29] who described a GTP-dependent phosphoenolpyruvate carboxykinase (PCK) from *T. kodakarensis*. PCKs have been historically classified into GTP- and ATP-dependent enzymes, with ATP-dependent enzymes described in bacteria, yeast, plants, and some eukaryotic parasites while the GTP-dependent enzyme being found in mammals and some other eukaryotes [30]. Interestingly, transcription of the gene encoding PCK was upregulated when *T. kodakarensis* cells were grown under gluconeogenic (with pyruvate and peptides as the carbon sources) rather than under glycolytic conditions (using starch as the carbon source). DNA microarray analysis showed that the same is true in *P. furiosus *when cells are grown on peptides and a sugar (maltose) [13]. As shown in Figure 3, PCK catalyzes the first step of gluconeogenesis, the decarboxylation of oxaloacetate to yield phosphoenolpyruvate and CO_2_. Hence we propose that during growth of *P. furiosus *by peptide fermentation, the function of the five ACS-G isoenzymes containing the *β*-G subunit is to provide GTP for the gluconeogenic-related PCK reaction. The ATP-independent generation of GTP by the ACS-G isoenzymes could also provide GTP for the archaeal translational machinery [31].

These data therefore suggest that all ten isoenzymes have physiological relevance in *P. furiosus*. That they all are functional is supported by DNA microarray data, which show that all ten genes are expressed under both gluconeogenic and glycolytic growth conditions and there appears to be little if any differential expression between the two growth modes for any of these genes [13]. The one exception is that the gene encoding the *α*-subunit of ACS2 (PF0532), which forms an operon with the genes encoding IOR, is up-regulated during growth on peptides. Proteomic analyses of *P. furiosus *grown on maltose and peptides also showed that all ten subunits of the ACS isoenyzmes are detected during fractionation of a cytoplasmic extract [14]. Moreover, immunoprecipitation analyses of *T. kodakarensis *showed that the *β*-G and the *β*-A subunits associated with multiple *α*-subunits [18]. Taken together, these data show that the ten ACS isoenzymes are constitutively produced and are able to generate both ATP and GTP from the wide variety of coenzyme-A derivatives produced during peptide fermentation. The same appears to be true for all members of the Thermococcales since, like *P. furiosus,* their genomes also contain genes encoding two *β*-subunits and five *α*-subunits of ACS.

**Figure 4:**
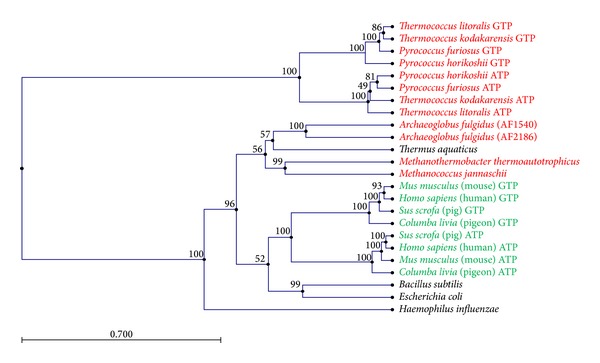
Phylogenetic tree of ACS *β* subunits. The unweighted pair group method of arithmetic mean (UPGMA) tree is shown. A neighbor-joining tree (data not shown) also shows similar relationships for the vertebrate and Thermococcales subunits with a few changes for the remaining species. Archaea are highlighted red, Bacteria are in black and Eukarya in green. Tree and Bootstrap values were generated using CLC Main Work Bench 6 with 100 resamples.

Homologs of ACS, known as succinyl-CoA synthetases (SCS), have been characterized from eukaryotes as well as bacteria [32, 33]. As with the *P. furiosus *enzymes, their nucleotide specificity is determined by their *β* subunits [34], although these differ from those in the ACSs of the Thermococcales as they contain an additional ligase domain [26, 35]. A phylogenetic tree of the *β*-subunits of the ACSs and SCSs is shown in Figure 4. It is striking that those of the Thermococcales form one clade while all other *β*-subunits fall into a second widely divergent clade. Each clade contains subunits that have a preference for one or the other purine nucleotide, indicating that they each arose in each clade by independent gene duplications. Interestingly, the ACSs that are found in Archaea that are not members of the Thermococcales, such as those in some methanogens and in the hyperthermophilic sulfate-reducer *Archaeoglobus fulgidus*, fall within a distinct branch within the eukaryote/bacterial clade of the tree rather than in the Thermococcales branch (Figure 4). Clearly, the *β*-subunits found in the ACSs of the Thermococcales are very distinct from those in all other ACSs/SCSs found throughout biology, including those in other hyperthermophilic archaea. Why and how this occurred are beyond the scope of this paper but are interesting subjects worthy of additional study.

## Figures and Tables

**Table 1:** Kinetic parameters of ACSx-A isoforms with CoA derivatives.

Enzyme	Substrate	*K*_*m*_ (*μ*M)	*k*_cat_/*K*_*m*_ (*μ*M^−1^·s^−1^)
ACS1-A	acetyl-CoA	137 ± 91	0.140
ACS2-A	acetyl-CoA	350 ± 2	0.114
ACS2-A	isovaleryl-CoA	466 ± 119	0.109
ACS2-A	phenylacetyl-CoA	3320 ± 1320	0.150
ACS3-A	acetyl-CoA	474 ± 165	0.012
ACS4-A	acetyl-CoA	242 ± 46	0.119
ACS5-A	acetyl-CoA	620 ± 1.0	0.112
ACS5-A	isobutyryl-CoA	2730 ± 1080	0.083
ACS5-A	isovaleryl-CoA	820 ± 407	0.120

**Table 2:** Nucleotide kinetics of ACS1 isoforms.

Isoform	Nucleotide	*K*_*m*_ (*μ*M)	*k*_cat_/*K*_*m*_ (*μ*M^−1^·s^−1^)
ACS1-A	ATP	132 ± 19	0.14
ACS1-A	GTP	223 ± 58	0.01
ACS1-G	ATP	342 ± 63	0.011
ACS1-G	GTP	87 ± 7	0.144
